# Hypoparathyroidism and Fahr’s Syndrome: A Case Series

**DOI:** 10.7759/cureus.40502

**Published:** 2023-06-16

**Authors:** Soumiya Berrabeh, Najoua Messaoudi, Ouafae Elmehraoui, Imane Assarrar, Ikram Karabila, Anouar Jamal, Nabila Zeryouh, Siham Rouf, Hanane Latrech

**Affiliations:** 1 Department of Endocrinology-Diabetology and Nutrition, Mohammed VI University Hospital Center, Oujda, MAR; 2 Department of Endocrinology-Diabetology and Nutrition, Faculty of Medicine and Pharmacy, Mohamed First University, Oujda, MAR; 3 Department of Endocrinology-Diabetology and Nutrition/Laboratory of Epidemiology, Clinical Research and Public Health, Faculty of Medicine and Pharmacy, Mohamed First University, Oujda, MAR

**Keywords:** fahr’s disease, neurological disorders, phosphocalcic metabolism, dysparathyroidism, intracerebral calcifications, basal ganglia

## Abstract

Fahr’s syndrome is defined by the presence of striato-pallido-dentate calcifications. It is a rare entity with clinical polymorphism, and it occurs in patients with dysparathyroidism, especially those with hypoparathyroidism. It must be distinguished from Fahr’s disease (FD), which is defined by the presence of intracerebral calcifications without phosphocalcic metabolism abnormality.

In this paper, we report the particulars of five patients diagnosed with Fahr’s syndrome revealed by neurological and cognitive disorders, seizures, and abnormal movements associated with tetany crisis. In all cases, brain imaging and biological examinations led to the diagnosis of Fahr’s syndrome related to hypoparathyroidism. The evolution was favorable after treatment.

Fahr’s syndrome is a rare and serious condition for which treatment is simple and effective. Our observations shed light on the necessity of evaluating phosphocalcic metabolism and exploring cerebral calcifications in patients with neurological disorders.

## Introduction

Hypoparathyroidism (HP) is a rare metabolic disorder that results in hypocalcemia, in which parathyroid hormone (PTH) secretion is inefficient in mobilizing calcium from bones and reabsorbing it by kidneys and intestines [1,2]. Anterior cervical surgery is the most frequent cause of acquired HP followed by autoimmune HP in adults. The duration, severity, and rate of the development of hypocalcemia determine the clinical presentation. A variety of organs can be affected by calcifications, more frequently kidneys, but also joints, eyes, skin, vessels, and, rarely, intracerebral calcifications. The latter is responsible for Fahr’s syndrome (FS) [3].

Fahr’s syndrome was described in 1930 by Theodor Fahr. Before that a plethora of descriptive terms were used to describe this neuro mineral disorder, with a total of 35 terms, resulting in considerable confusion as to which cases constitute this disease [4,5].

FS associates symmetrical calcifications of areas of the brain that control movements, including the basal ganglia (BG), thalamus, and others; neuropsychiatric symptoms; and hypofunction of the parathyroid gland [6]. The main symptoms of bilateral basal ganglia calcification (BGC) include extrapyramidal and cerebellar disorders, cognitive impairment, epileptic seizures, and psychiatric changes [3]. BGC has been established as a possible outcome of HP, and its prevalence, demonstrated in the HP cohorts, varied significantly from 12% up to 74% [1,3]. Currently, computed tomography (CT) is the most valuable method for diagnosis.

Fahr’s syndrome must be distinguished from Fahr’s disease, which corresponds to calciﬁcations of the basal ganglia without abnormalities of phosphocalcic metabolism and which may be genetic or sporadic [7].

The treatment of FS is mainly symptomatic; however, there is no specific treatment limiting the progression of calcifications in the basal ganglia. Conversely, early treatment can prevent calcifications and neurophysiological disorders, especially in HP [3,6]. Several cases have been reported with a variety of onset modes [6]. This illustrates the benefit of performing a phosphocalcic workup in case of intracerebral calciﬁcations and neurological or neuropsychiatric disorders poorly controlled by usual treatments (e.g., antiepileptic drugs, antidepressants, hormone replacement therapy).

We report five cases of Fahr’s syndrome related to primary hypoparathyroidism (PHP) with polymorphic modes of onset. We will discuss the clinical, radiological, and biological profiles of each patient. This case series has been reported following the SCARE criteria [8].

## Case presentation

Case 1

**Figure 1:**
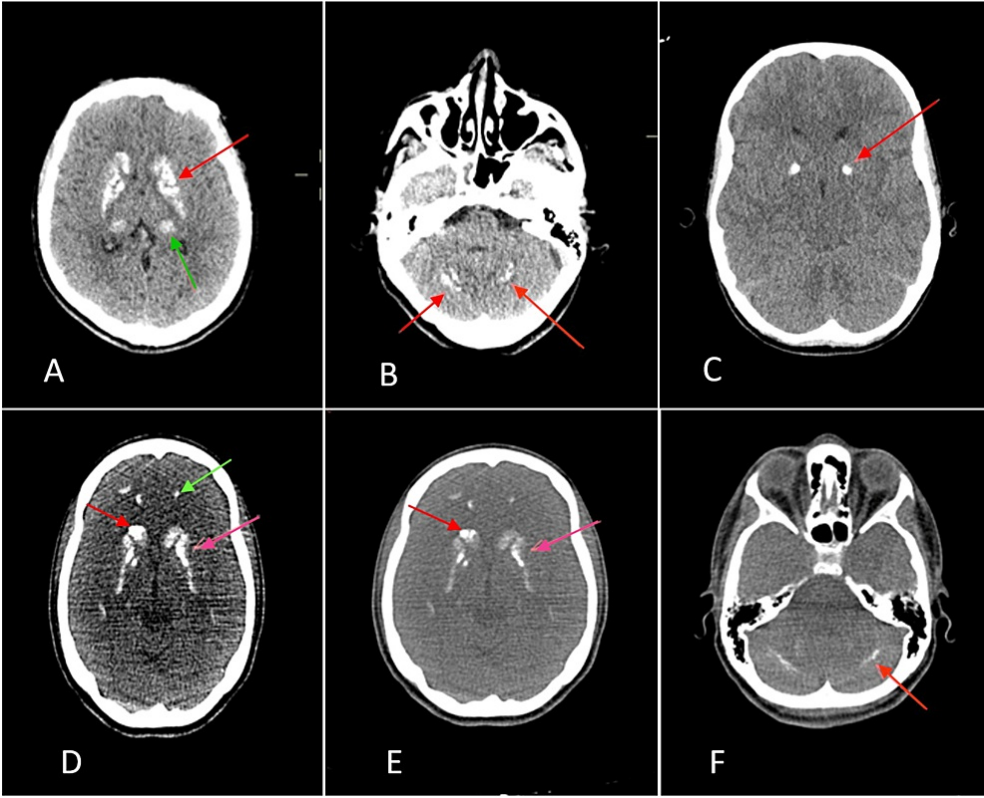
Brain CT scan showing bilateral calcifications of the basal ganglia A. Calcifications of the thalami (green arrow) lenticular and caudate nuclei (red arrow) B. Calcifications of the dentate nuclei (red arrow) C. Calcifications of the lenticular nuclei (red arrow) D. Calcifications of the caudate nuclei (red arrow), subcortical structures (green arrow), and lenticular nuclei (pink arrow) E. Calcifications of the lenticular (pink arrow) and caudate nuclei (red arrow) F. Calcifications of the dentate nuclei (red arrow)

A 16-year-old female presented to the emergency room (ER) with generalized tonic-clonic seizures. She had a history of recurrent crises associated with cognitive impairment since the age of 14. A brain CT scan revealed bilateral and symmetrical calcifications of the lenticular and dentate nuclei, raising suspicion of FS (Figure 1). The phosphocalcic assessment showed severe hypocalcemia, hyperphosphatemia, a low bio-intact PTH level, and a low 25(OH) vitamin D level. Magnesium, liver, and kidney function tests were normal. The electrocardiogram (ECG) showed a prolonged QT interval. The ophthalmological examination revealed a bilateral polar cataract. The diagnosis of FS related to HP was made. Intravenous correction of hypocalcemia was instituted enabling the disappearance of the seizures, followed by oral substitution treatment associating calcium (60 mg/kg/day) and vitamin D (1-alpha-OH-vitamin D3 1 µg/day). One month later, we discontinued calcium supplementation and insisted on calcium-rich foods intake. A clinical and biological improvement was noted during the follow-up (Table 1).

**Table 1:** Clinical and biological characteristics of the patients and follow-up F: female, PHP: primary hypoparathyroidism, PTH: parathyroid hormone, NV: normal value

			Case 1	Case 2	Case 3	Case 4	Case 5
Gender			F	F	F	F	F
Age (Years)			16	22	12	22	14
Family cases			No	No	No	No	No
Clinical symptoms			Generalized tonic-clonic seizures associated with cognitive impairment	Tetanic seizures	Tetanic seizures	Tetanic seizures with abnormal movements	Seizure, mood disorders
Cerebral CT scan			Bilateral and symmetrical calcifications of the lenticular and dentate nuclei	Bilateral and symmetrical calcifications of the caudate and lenticular nuclei	Bilateral and symmetrical calcifications of the pallidum of the lenticular and dentate nuclei	Bilateral and symmetrical calcifications of the lenticular nucleus and the thalamus	Bilateral and symmetrical calcifications of the white matter, semi-oval center, thalami, caudate, dentate, and lenticular nuclei
Calcemia (mg/l)		NV: 84–102	53	60	69	45	62.7
		Technique: spectrophotometry					
Phosphatemia (mg/l)	NV: 23–47		88	80	62	90	83.5
	Technique: spectrophotometry						
Bio-intact PTH (1-84) pg/ml	NV: 15–57		5.50	6.3	10.5	5	4.66
	Technique: chemiluminescence CMIA						
25(OH) vitamin D (ng/ml)	NV: > 30		9.3	8.4	10	10	27
	Technique: CMIA						
24H calciuria (mg/Day)	NV: 100–300		87	79	20 (0.68 mg/kg/day)	45	8.4 (0.2 mg/kg/day)
	Technique: spectrophotometry						
Etiology			PHP	PHP	PHP	PHP	PHP
Treatment			Calcivitamin D therapy	Calcivitamin D therapy	Calcivitamin D therapy	Calcivitamin D + anti-epileptic therapy	Calcivitamin D therapy
Follow-up (duration)			Clinical and biological improvement (5 years)	Clinical and biological improvement (3 years)	Clinical and biological improvement (6 months)	Clinical and biological improvement (2 years)	Clinical and biological improvement (1 month)

Case 2

A 22-year-old female patient was brought to the ER because of tetanic seizures. The first biological assessment found profound hypocalcemia. A cerebral CT scan showed bilateral and symmetrical calcifications of the caudate and lenticular nucleus. Etiologic investigations of hypocalcemia confirmed the diagnosis of primary hypoparathyroidism. The patient was treated with vitamin D and calcium supplementation. A clinical and biological improvement was observed during the follow-up (Table 1).

Case 3

A young female patient of 12 years old consulted the ER for tetanic seizures. The biological workup revealed deep hypocalcemia. Primary hypoparathyroidism (probably autoimmune PHP) was confirmed: low bio-intact PTH, 25(OH) vitamin D and 24H calciuria levels, and hyperphosphatemia. Magnesium, liver, and kidney function tests were normal. The ECG did not show any QT interval abnormality and the ophthalmologic evaluation was normal. The brain CT scan revealed bilateral and symmetrical calcifications of the pallidum of the lenticular nuclei and dentate nuclei. Thus, PHP-related Fahr’s syndrome was retained. The patient received a progressive correction of hypocalcemia using intravenous treatment, then oral treatment that consisted initially of calcium 1 g/d and vitamin D (1-alpha-OH-vitamin D3) 0.25 µg/d, and then only vitamin D. During follow-ups, the patient reported clinical improvement with normalization of blood calcium levels (Table 1).

Case 4

A 22-year-old woman was admitted to the Department of Neurology for tetanic seizures with abnormal movements. She suffered from this condition and extremities tingling for the last year. She had a history of epilepsy for three years and was put under sodium valproate. A routine workup was performed during her stay that showed severe hypocalcemia. Our medical team examined the patient and prescribed further assessment. Therefore, the diagnosis of primary hypoparathyroidism (probably autoimmune PHP) was established based on low levels of PTH and 25(OH) vitamin D and hyperphosphatemia. Magnesium, liver, and kidney function tests were normal. The ECG showed a prolongation of the QT interval. The brain CT scan showed bilateral and symmetrical calcifications of the lenticular nucleus and the thalami. Intravenous correction of hypocalcemia allowed the disappearance of the tingling and the seizures. Then, a substitution treatment combining calcium (3 g/day) and vitamin D (1-alpha-OH-vitamin D3 1 µg/day) was instituted. During follow-up, the patient remained in good condition and blood calcium levels were normal (Table 1).

Case 5

A 14-year-old female patient was admitted to the ER for seizures. A cerebral CT scan revealed diffuse intracerebral calcifications. A blood workup found the following: hypocalcemia, a decreased 1-84 PTH level, insufficient 25(OH) vitamin D, 24H hypocalciuria, and hyperphosphatemia. Magnesium level, and liver and kidney function tests were normal. The ECG was normal. The brain scan showed diffuse calcifications in all of the basal ganglia (Figure 1). The patient received a progressive correction of hypocalcemia by intravenous treatment, then oral treatment: calcium 1 g/d and vitamin D (1-alpha-OH-vitamin D3) 2 µg/d, and then treatment by only vitamin D. Clinically, the patient showed signs of recovery with normalized calcium levels during follow-ups (Table 1).

## Discussion

Fahr’s syndrome (FS) or secondary calcifications is defined radiologically by the presence of striato-pallido-dentate, non-arteriosclerotic, bilateral, and symmetric calcifications [5]. It is a rare condition with a prevalence of less than 1/1,000,000 individuals [1,3].

Histochemical analysis of post-mortem patients with Fahr’s syndrome found basophilic mineral deposits in the vascular walls (arterial, capillary, and venous vessels) and the perivascular spaces, with calcium as the principal element. This may explain its radiological appearance. In addition, other minerals were also described, including traces of aluminum, arsenic, magnesium, phosphorus, and copper [9-12]. The physiopathological mechanism responsible for these deposits remains poorly understood.

The high cerebral blood flow and metabolic rate of the basal ganglia, particularly the globus pallidus, could be the reason for them being a soft spot for calcifications. It also makes them prone to hypoxia, high mucopolysaccharide, and phosphorus deposits. Moreover, their periventricular placement allows the exchange of calcium and phosphate between the cerebrovascular fluid and the cerebral parenchyma [13,14]. However, the basal ganglia are targets for other mineral and non-mineral contents [5,14]. In Fahr’s disease, some authors have suggested an exaggeration of the normal process of calcium or iron deposits in the basal ganglia [15].

Fahr’s syndrome is usually difficult to suspect clinically because of the broad spectrum of the manifestations and its polymorphism; besides, the anatomo-clinical correlation is not always obvious. It may involve intellectual delay or deterioration, character disorders, and sometimes even delirious episodes [16]. An association of symptoms attributed to different areas of the central nervous system (CNS) is also common [1]. We found in our series and also in the literature that epileptic seizures (almost in 40% of the cases, mainly generalized tonic-clonic seizures) and tetany were the most frequent symptoms at the onset of the disease, followed by movement disorders and neuropsychiatric symptoms or a combination of the previous symptoms [6]. As for clinico-radiological correlation, one of our patients presented severe neuropsychological and behavior disorders correlated radiologically with extended calcifications. This observation is described in the literature, in which the severity of the Fahr syndrome phenotype is associated with the extent of calcifications in the basal ganglia, white matter, or other areas. These neurological and neuropsychological disorders could be explained with the BG being included in the cortico-subcortical systems and its connection to prefrontal and frontal areas [17,18].

Other movement disorders reported in the literature but not found in our patients include Parkinson’s syndrome in 52% of cases and hyperkinetic movement disorder in about 35% [19]. Also, FS may remain asymptomatic in some cases [16,20,21].

The diagnosis of Fahr’s syndrome is usually delayed, mainly in patients with primary hypoparathyroidism compared to other causes [6]. In our patients, the average diagnostic delay was 7.4 years with extremes between 4 and 10 years. The polymorphic and nonspecific clinical presentation of intracerebral calciﬁcations explains the delay in diagnosis and management. In some cases, hypocalcemia is revealed by the diagnosis of FS. Moreover, several reported cases of FS have been clinically asymptomatic but detected incidentally on radiological examinations (15-20% of cases) [6,22].

FS is caused by three main etiologies: hypoparathyroidism, pseudo-hypoparathyroidism, and hyperparathyroidism. According to a review of 223 cases, hypoparathyroidism was the most common cause, which is in accordance with our results, where all our patients have FS related to hypoparathyroidism. Its biological diagnosis was based on the association of hypocalcemia, hyperphosphatemia, hypocalciuria, and decreased serum PTH levels. Idiopathic/familial hypoparathyroidism is in fact the most common etiology. Postsurgical hypoparathyroidism represents the second-most common cause, with a mean time of diagnosis almost 30 years after thyroidectomy [4,6]. Pseudo-hypoparathyroidism is less frequent and often of familial origin. It has a similar clinical and biological profile to hypoparathyroidism, except for normal PTH levels due to peripheral resistance to the hormone [23]. Hyperparathyroidism has been exceptionally reported as a cause of this syndrome [16,19,24]. In our series, we ruled out both etiologies.

A Cerebral CT scan is the gold standard for diagnosing intracerebral calciﬁcations [13,20,21]. As for the distribution pattern, calcifications are typically bilateral and symmetrical, most commonly localized in our series in the thalami and dentate and lenticular nuclei, but also in the caudate nuclei, the semi-oval center, and white matter. However, the literature reported 72% of calcifications localized in the cerebellum, 50% in white matter, 38% in the thalami, and 23% in other areas [6,25]. In our series, we used cerebral CT scans for all patients to diagnose BGCs (Table 1).

Cerebral magnetic resonance imaging (MRI) is not commonly used for brain calcifications. It usually shows hyperintense signals in T1 and T2 weighted images involving the same areas [26]. However, functional imaging provides new insights into the physiopathological mechanisms responsible for FS as well as a clear study of these calcifications. Indeed, some studies have found perfusion abnormalities in calciﬁed regions on single photon emission computed tomography (SPECT) scans, whereas positron emission tomography (PET) scans found no signiﬁcant difference [5]. Thus, it would be ideal to combine the different imaging methods for a better study of the functions of the central nucleus [6].

Considering the heterogenicity of the clinical presentations, other causes of intracerebral calciﬁcations should be discussed such as endocrinopathies, systemic diseases, celiac disease, infections, primary or secondary calciﬁed brain tumors, and other various diseases (chronic renal failure, vitamin D intoxication, etc.) [17,18,27]. In these cases, the calciﬁcations are not bilateral nor symmetrical and are not localized in the central grey nuclei, as observed in FS. However, the causal association remains difficult to establish in the absence of clear physiopathological elements [28].

In the case of intracerebral calcifications without abnormalities of phosphocalcic metabolism, Fahr’s disease (idiopathic or primary calcifications) must be evoked. The confirmation of FD is based on the Moskowitz criteria, which associates intracerebral calcifications with neurological symptoms and familial character without abnormalities of phosphocalcic metabolism [29]. In FD, the extrapyramidal syndrome is the main manifestation, together with cerebellar and pyramidal syndromes and cognitive disorders with progressive evolution to dementia. The age of discovery is between 40 and 50 years with male predominance. Some patients are symptomatic even in the absence of lesions on CT scans; this suggests that macroscopic calciﬁcations are not the primum movens [30,31].

The treatment of FS includes calcivitamin D therapy and etiologic identification [16,24]. Early treatment can prevent calcifications and neurophysiological disorders, especially in hypoparathyroidism. However, there is no specific treatment limiting the evolution of calcifications in the basal ganglia. Other treatments have also been tested, including speciﬁc intracerebral calcium canal blockers that were unsuccessful and disodium etidronate that affected clinical signs, which were confirmed in a double-blind study and compared with a placebo [32]. In addition to treatment, the importance of long-term clinical and biological monitoring of these patients must be emphasized.

In general, FS has a good prognosis in contrast to the severity of its symptoms. Correction of phosphocalcic metabolism disorders often leads to significant regression of the symptomatology [16,20]. In our patients, calcivitamin D therapy allowed notable clinical and biological improvement.

Our series of five patients highlights the importance of assessing phosphocalcic metabolism in the case of neurologic and neuropsychiatric symptoms. Also, it demonstrates the importance of appropriate treatment in alleviating symptoms and assuring good control of the disease. However, these series remain limited in the number of patients. Larger studies are needed to further support our findings.

## Conclusions

Fahr’s syndrome remains a rare and serious anatomo-clinical and radiological entity complicating non-diagnosed or poorly treated chronic hypocalcemia, for which treatment is simple and effective. Our observations emphasize the need to search for abnormalities of phosphocalcic metabolism and cerebral calcifications in any patient presenting neuropsychiatric disorders. FS should be distinguished from Fahr’s disease, where biological workup is normal and genetic assessment is necessary.
